# Patterns of Use of Smartphone-Based Interventions Among Latina Breast Cancer Survivors: Secondary Analysis of a Pilot Randomized Controlled Trial

**DOI:** 10.2196/17538

**Published:** 2020-12-08

**Authors:** Sharon H Baik, Laura B Oswald, Joanna Buscemi, Diana Buitrago, Francisco Iacobelli, Alejandra Perez-Tamayo, Judith Guitelman, Frank J Penedo, Betina Yanez

**Affiliations:** 1 Department of Medical Social Sciences Northwestern University Feinberg School of Medicine Chicago, IL United States; 2 Department of Supportive Care Medicine City of Hope Duarte, CA United States; 3 Department of Health Outcomes and Behavior Moffitt Cancer Center Tampa, FL United States; 4 Department of Psychology DePaul University Chicago, IL United States; 5 Department of Computer Science Northeastern Illinois University Chicago, IL United States; 6 Department of Surgery University of Illinois at Chicago Chicago, IL United States; 7 ALAS-WINGS The Latina Association for Breast Cancer Chicago, IL United States; 8 Department of Psychology University of Miami Coral Gables, FL United States

**Keywords:** breast cancer, cancer survivorship, Hispanics/Latinas, eHealth, psychosocial intervention, mobile phone

## Abstract

**Background:**

Latina breast cancer survivors experience poorer health-related quality of life (HRQoL), greater symptom burden, and more psychosocial needs compared to non-Latina breast cancer survivors. eHealth platforms such as smartphone apps are increasingly being used to deliver psychosocial interventions to cancer survivors. However, few psychosocial eHealth interventions have been developed specifically for Latina breast cancer survivors. Further, little is known about how Latinas, in general, engage with eHealth interventions and whether specific participant characteristics are associated with app use in this population. We evaluated the use of 2 culturally informed, evidence-based smartphone apps for Latina breast cancer survivors—one that was designed to improve HRQoL and reduce symptom burden (*My Guide*) and the other to promote healthy lifestyle behaviors (*My Health*).

**Objective:**

The objectives of our study were to explore the patterns of use of the *My Guide* intervention app and *My Health* attention-control app among Latina breast cancer survivors.

**Methods:**

Eighty Latina breast cancer survivors were randomized to use the *My Guide* or *My Health* app for 6 weeks. Assessments were collected at baseline (T1), immediately after the 6-week intervention (T2), and 2 weeks after T2 (T3). Specific study outcomes included subdomains of HRQoL, symptom burden, cancer-specific distress, cancer-relevant self-efficacy, and breast cancer knowledge.

**Results:**

On average, participants used their assigned app for more than 1 hour per week. Sociodemographic or psychological characteristics were not significantly associated with app use, except for employment status in the *My Health* group. Content related to common physical and emotional symptoms of breast cancer survivors as well as recommendations for nutrition and physical activity were most frequently accessed by *My Guide* and *My Health* participants, respectively. Lastly, clinically meaningful improvements were demonstrated in breast cancer well-being among low app users (ie, <60 minutes of use/week) of *My Guide* and social well-being among high app users (ie, ≥60 minutes of use/week) of *My Health*.

**Conclusions:**

The favorable rates of participant use across both apps suggest that Latina breast cancer survivors are interested in the content delivered across both *My Guide* and *My Health*. Furthermore, since sociodemographic variables, excluding employment status, and baseline HRQoL (psychological variable) were not related to app use, *My Guide* and *My Health* may be accessible to diverse Latina breast cancer survivors.

**Trial Registration:**

ClinicalTrials.gov NCT03645005; https://clinicaltrials.gov/ct2/show/NCT03645005

## Introduction

Breast cancer is the most prevalent cancer and the leading cause of cancer-related deaths among Latina women in the United States, with an estimated 24,000 new cases diagnosed and 3200 deaths expected annually [1]. Currently, there are more than 200,000 Latina breast cancer survivors living in the United States [2] and this number is expected to continue increasing. Latina breast cancer survivors experience disparities in survivorship outcomes compared to non-Latina breast cancer survivors, including lower health-related quality of life (HRQoL), greater symptom burden, and greater unmet psychosocial needs [3-11]. These factors are associated with poorer health outcomes [12,13] and must be addressed to promote optimal long-term survivorship for Latina breast cancer survivors. However, Latinas are underrepresented in cancer research [9,14] and although a number of psychosocial interventions have demonstrated efficacy for improving HRQoL outcomes among breast cancer survivors [13,15,16], few interventions have been developed specifically for Latina breast cancer survivors [3,9,17]. Further, psychosocial interventions may be more effective when culturally and linguistically tailored to a particular racial/ethnic group [18]. Specifically, some Latina cultural values and beliefs (eg, familism, fatalism, Marianismo) [19,20] may impact health behaviors and outcomes and thus should be considered as intervention approaches that may need to be adapted. In general, culturally adapted interventions have demonstrated moderate-to-large effects [21].

eHealth platforms are increasingly being used to deliver psychosocial interventions to general cancer survivors and have demonstrated positive effects related to psychosocial outcomes and lifestyle behaviors [22,23]. Research shows that Latinas own smartphones and surf the internet using their mobile devices at similar or higher rates than other racial/ethnic groups [24]. Therefore, smartphone-based apps provide an innovative opportunity to implement culturally informed, technology-based, and evidence-based psychosocial interventions for this population to overcome barriers to accessing care and resources and reduce participant and provider burden, and they are more easily scalable than traditional in-person intervention delivery methods. Prior research has demonstrated that physical and psychological outcomes in eHealth interventions are influenced by different measures of adherence or usage (eg, number of logins, modules completed, time spent on app) [25], and higher use of web-based interventions among cancer survivors is associated with specific user characteristics such as having low social support and high illness burden or working and receiving radiation therapy [26,27]. However, little is known about how Latina breast cancer survivors engage with eHealth interventions or what participant characteristics are associated with eHealth use in this population.

Our team developed and evaluated 2 culturally informed and evidence-based smartphone apps for Latina breast cancer survivors in a 6-week pilot randomized controlled trial. The intervention app, *My Guide,* was designed to improve HRQoL, reduce symptom burden, and reduce cancer-specific distress, and the attention-control app, *My Health,* was designed to promote general health and healthy lifestyle behaviors [28]. Results demonstrated that Latina breast cancer survivors across both study conditions reported temporary decreases in symptom burden and improved breast cancer well-being over time, although there were no differential effects between the apps [29]. The primary objective of this secondary analysis was to report patterns of participant use of the *My Guide* and *My Health* smartphone apps over the course of the pilot randomized controlled trial. More specifically, we provide data on the frequency of specific domains accessed within the *My Guide* and *My Health* apps and the average total duration of app use. Determining the most commonly accessed and viewed topics is clinically important, as it can help inform future refinement of the study apps as well as in-person psychosocial interventions designed to address the most important cancer topics for this understudied population. Furthermore, given the limited research in this area, we explored whether participant characteristics were related to app use as well as whether app use was related to study outcomes over time.

## Methods

### Study Participants and Design

Women were eligible to participate in the study if they were diagnosed with nonmetastatic stage 0-III breast cancer, had completed active treatment for breast cancer (excluding endocrine therapy), were within 2-24 months of completing treatment, were at least 21 years old, able to read and speak English or Spanish, and able to provide informed consent. Women were excluded if they had a diagnosis of another cancer, serious psychiatric disorder (eg, bipolar disorder, substance dependence), or life-threatening illness (eg, end-stage kidney disease). Study recruitment included advertisements and physician referrals from 2 academic medical centers in the Chicago area and ALAS-WINGS, a community-based organization that serves Latina women with breast cancer.

Eligible participants provided informed consent and completed a sociodemographic questionnaire and psychosocial assessment at baseline (T1) in their preferred language (English or Spanish). Follow-up assessments were completed at postintervention (6 weeks from baseline, T2) and 2 weeks after the intervention (8 weeks from baseline, T3). After providing informed consent, participants were randomized 1:1 to receive either *My Guide* or *My Health* smartphone-based app for 6 weeks, and participants were instructed to use their assigned app for 2 hours per week. Participants continued to have access to their assigned app from T2 to T3, but we did not analyze their usage data (ie, time spent on app, webpages clicked), and reinforcement messages or calls for adherence were not provided during this time.

App use for *My Guide* and *My Health* was supported by trained bilingual telecoaches using a stepped-care approach. Specifically, telecoaching calls were provided to all participants who focused on enhancing their adherence to using their assigned app and addressed any issues or barriers to using the app. All participants received telecoaching calls during the first 2 weeks as well as during the final sixth week. Subsequent telecoaching calls during the third, fourth, and fifth weeks were only made to participants who did not use their app for at least 90 minutes per week (threshold), whereas reinforcing text messages were sent to participants who used their app for 90 minutes or more. Telecoaching was brief (~15 minutes) and delivered using motivational interviewing and goal setting to encourage app use during the study period. Telecoaching calls were audio-recorded and reviewed weekly by a licensed clinical psychologist to ensure fidelity. A threshold of 90 minutes of weekly app use was determined by other web-based studies that also focused on the delivery of symptom management for patients with cancer [30,31]. On average, *My Guide* and *My Health* app users received 3.72 and 4.10 telecoaching calls, respectively, over the 6-week study period. Further details of the study design, development, and protocol are published elsewhere [28]. All study procedures and assessments were approved by the Institutional Review Board at Northwestern University prior to study recruitment.

### Study Apps

Both smartphone apps were developed by the Center for Behavioral Intervention Technologies at Northwestern University Feinberg School of Medicine [28]. The content for *My Guide* and *My Health* was culturally informed by Latina cultural values and beliefs (eg, familism, fatalism, Marianismo) [19,20] and developed in collaboration with the Latina Breast Cancer Association, a community partner. Both apps were developed in English and Spanish so that they were each delivered in the participant’s preferred language, and all content was available in audio format to address concerns about low literacy.

The *My Guide* app was designed to improve HRQoL and reduce symptom burden among Latina breast cancer survivors. The intervention content was based on models of stress and coping [32-34], prior research on psychosocial adjustment to cancer [35,36], and preliminary results suggesting that self-efficacy in patient-provider communication, cancer-related knowledge, stress management, and social support can improve HRQoL and symptom burden among Latina breast cancer survivors [3,19,35,37-39]. Specifically, the content focused on improving psychosocial adaptation during cancer survivorship, coping with side effects from treatment, stress management, social support, and breast cancer–related knowledge. *My Health*, the attention-control app, was designed to improve health-promoting behaviors and provided general recommendations for nutrition and exercise, prevention of common chronic illnesses, and other healthy lifestyle behaviors. The control content was based on similar studies of psychosocial interventions among cancer survivors with active controls [31]. Each app comprised of 6 content domains or distinct topic areas (see Multimedia Appendix 1 for a brief summary of each domain and its related content) as well as a media component (“*Listen and Learn*”) consisting of videos and audio recordings that were incorporated and complemented information throughout the domains. For example, *My Guide* included informational videos from experts on breast health and common side effects, stories from other cancer survivors, and audio programs of relaxation exercises, whereas *My Health* included videos about healthy eating and lifestyle behaviors. Both apps were designed to be self-guided, such that participants had complete access to all the domains and could freely access any content based on their interest, at their own pace, and at any time.

### Measures

All measures were provided in the participants’ preferred language of English or Spanish.

#### Sociodemographic and Cancer-Specific Characteristics

At baseline, participants completed a self-report sociodemographic questionnaire, which included information such as age, ancestry (Mexican vs other), language preference, highest education, annual household income, employment status, and marital status. Additionally, participants self-reported cancer-specific characteristics, which were verified by medical chart review, including stage of disease and type of treatment(s) received.

#### App Use

Both apps tracked participant use in minutes per week throughout the study period, from which the total 6-week app use was computed by summing the 6 weekly use times. Average weekly use was also computed to help with interpretation (ie, total app use in minutes divided by 6 weeks). Additionally, all actions taken within each app (ie, click data) were tracked for each participant. The log files included the following data for each action: participant information (ie, unique identification number), app information (ie, English vs Spanish, *My Guide* vs *My Health*), and timestamp (date and time) of links clicked. From this data, we extracted information related to the frequency of visits to each webpage (links) in the app for each participant. We were then able to determine the most and least accessed content within each app.

#### HRQoL

The 36-item Functional Assessment of Cancer Therapy-Breast (FACT-B) [40] measures 5 HRQoL subdomains, namely, physical well-being, emotional well-being, social well-being, functional well-being, and additional breast cancer-related concerns. Respondents were asked to rate their level of agreement with statements regarding concerns over the past 7 days by using a 5-point Likert-type scale from 0 (not at all) to 4 (very much). Higher scores indicate better domain-specific well-being. The FACT-B has been validated in Spanish [41] and is extensively used among patients with breast cancer [40,41]. Additionally, at baseline, participants completed the rapid version of the Functional Assessment of Cancer Therapy-General (FACT-G7), a valid and reliable 7-item measure of the most prominent HRQoL concerns among cancer survivors [42].

#### Symptom Burden

The 25-item Breast Cancer Prevention Trial questionnaire [43] consists of 25 common breast cancer–related symptoms. Respondents rated their level of discomfort with each symptom during the past 4 weeks by using a 5-point Likert-type response scale from 0 (not at all) to 4 (extremely). Total scores range from 0 to 100, with higher scores indicating higher breast cancer symptom burden.

#### Cancer-Specific Distress

The 15-item Impact of Events Scale [44] assesses the frequency of intrusive thoughts or avoidance following a stressful event, specifically cancer, and uses a response scale with 4 points, that is, 0 (not at all), 1 (rarely), 3 (sometimes), and 5 (often). Total scores range from 0 to 75, with higher scores indicating greater distress. The Impact of Events Scale has also been validated in Spanish [45].

#### Cancer-Relevant Self-Efficacy

The 12-item Communication and Attitudinal Self-Efficacy scale for cancer (CASE-cancer) [46] assesses one’s self-efficacy in emotional resilience, communication, and information seeking in the context of a cancer diagnosis. Respondents rated their confidence using various skills on a 4-point response scale from 1 (not at all) to 4 (extremely). Higher values indicate greater self-efficacy with possible values ranging from 12 to 48. Previous studies have used the CASE-cancer with Latina breast cancer survivors [46,47].

#### Breast Cancer Knowledge

The 16-item Knowledge about Breast Cancer questionnaire assesses general knowledge about breast cancer diagnosis and treatment. Respondents were asked to answer *true* or *false* to 16 statements. Correct responses were first summed and then divided by the total number of responses to compute an average correct response score. Total scores range from 0 to16, with higher scores reflecting better breast cancer knowledge [48]. This questionnaire was previously tested with a large sample of Spanish-speaking Latina breast cancer survivors [48] and used in the initial *My Guide* pilot study [28].

### Data Analyses Plan

We conducted linear regression analyses to evaluate whether sociodemographic characteristics (ie, age, language preference, education level, total household income, ancestry, marital status, employment status) or baseline psychological variables (ie, FACT-G7 baseline score) were related to total app use. Clinical significance was determined with *P*<.05, and marginal associations were reported with *P*<.10. Frequencies of total clicks (ie, pages viewed) were calculated for each domain and subdomain within each app, and we used descriptive statistics to describe notable patterns in click-level app use. Given the exploratory nature of these analyses and underpowered sample size to conduct inferential statistics within subgroups of app users, we focused on descriptive statistics to characterize study outcomes across time by high (ie, ≥60 minutes per week) versus low (ie, <60 minutes per week) use of the *My Guide* or *My Health* app. Established minimally important differences for the FACT-B subscales (ie, a minimum of 2 points) [49-51] were used to characterize changes in the HRQoL subdomains across time. The established minimally important differences are not available for other study outcomes.

## Results

### Sample Characteristics

Latina breast cancer survivors (N=80) were enrolled and randomized to use *My Guide* or *My Health*. However, 2 participants (one from each condition) were withdrawn due to technical issues, resulting in a total of 78 Latina breast cancer survivors analyzed. Table 1 presents the demographic characteristics of the sample. Overall, participants had a mean (SD) age of 52.54 (11.36) years, and most were born outside the United States (55/78, 71%), of Mexican ancestry (50/78, 64%), and with Spanish as their preferred language (50/78, 64%). There were no significant differences in the sociodemographic and clinical characteristics between study conditions (*P*>.05).

**Table 1:** Baseline sociodemographic and clinical characteristics.

Characteristics		Full sample (n=78)	*My Guide* app users (n=39)	*My Health* app users (n=39)
**Age (years)**				
	Mean (SD)	52.54 (11.36)	53.52 (11.25)	51.55 (11.53)
	Range	29-75	33-73	29-75
**Language preference, n (%)**				
	English	28 (36)	14 (36)	14 (36)
	Spanish	50 (64)	25 (64)	25 (64)
Mexican ancestry, n (%)		50 (64)	25 (64)	25 (64)
Born in the United States, n (%)		23 (30)	14 (36)	9 (23)
High school education or less, n (%)		42 (54)	23 (59)	19 (49)
Annual household income <US $25,000, n (%)		41 (53)	23 (59)	18 (46)
Employed, n (%)		34 (44)	17 (44)	17 (44)
Married or partnered, n (%)		50 (64)	23 (59)	27 (69)
**Stage of breast cancer, n (%)**				
	0	3 (4)	2 (5)	1 (3)
	I	28 (36)	14 (36)	14 (36)
	II	32 (41)	16 (41)	16 (41)
	III	11 (14)	5 (13)	6 (15)
	Did not report	4 (5)	2 (5)	2 (5)
Received chemotherapy, n (%)		45 (58)	21 (54)	24 (62)
Received radiation therapy, n (%)		55 (71)	28 (72)	27 (69)
FACT-G7^a^ baseline score, mean (SD)		19.947 (4.98)	20.053 (5.17)	19.842 (4.86)

^a^FACT-G7: Functional Assessment of Cancer Therapy-General 7 items.

### App Use

Latina breast cancer survivors used their assigned smartphone app for mean (SD) time of 478.15 (385.84) minutes over the 6-week study period. The mean (SD) weekly app use did not differ between *My Guide* (86.58 [66.08] minutes per week) and *My Health* (72.80 [62.57] minutes per week, t_76_=–0.95; *P*=.34). See Table 2 for the total app use for each week, average weekly use, and total use over the 6-week study period. A notable pattern across both apps is the reduced weekly app use during the third week followed by an increase during the subsequent week, presumably after receiving more telecoaching.

**Table 2:** Total time and average time of app usage.

Weeks	*My Guide* app users (n=39), time (minutes)		*My Health* app users (n=39), time (minutes)	
	Mean (SD)	Range	Mean (SD)	Range
Week 1	95.97 (102.72)	0-431	65.74 (73.99)	0-400
Week 2	90.51 (93.80)	0-426	86.33 (105.45)	0-376
Week 3	69.03 (69.87)	0-250	66.79 (72.89)	0-251
Week 4	87.26 (81.30)	0-350	73.26 (74.46)	0-264
Week 5	91.13 (82.30)	0-311	77.69 (89.06)	0-372
Week 6	85.59 (85.51)	0-318	67.00 (81.40)	0-378
Total usage (weeks 1-6)	519.49 (396.51)	0-1612	436.82 (375.43)	0-1551
Average weekly usage	86.58 (66.08)	0-269	72.80 (62.57)	0-259

### Predictors of App Use

There were no significant relationships between the sociodemographic characteristics or baseline HRQoL and total app use (all *P*>.10), except for employment status. Specifically, for *My Health*, participants who were employed used the app for a lesser duration than those who were unemployed (*β*=–.33; *P*=.04).

### Patterns of App Use

Table 3 presents the click-level data for each domain in each app. Click-level data for each subdomain is presented in Multimedia Appendix 2.

**Table 3:** Total number of clicks for each domain within each app.

App type, domains		n (%), Values
**My Guide app (n=6368)**		
	Managing My Emotions	1731 (27.18)
	Managing My Symptoms	963 (15.12)
	Managing My Health	784 (12.31)
	Breast Cancer Medications	318 (4.99)
	Family and Friends	608 (9.55)
	Community and Everyday Support	685 (10.76)
	Listen and Learn (Media)	1279 (20.08)
**My Health app (n=7167)**		
	Diet and Nutrition part 1	1339 (18.68)
	Diet and Nutrition part 2	1527 (21.31)
	Exercise	1391 (19.41)
	Preventing Diabetes and Heart Disease	573 (7.99)
	Lifestyle Behaviors	929 (12.96)
	Doctor’s Recommendations	999 (13.94)
	Media	409 (5.71)

#### My Guide App

Over the 6-week study period, *My Guide* participants clicked on a total of 6368 links or webpages of intervention content within the app. Participants most frequently accessed content within the *Managing My Emotions* domain (1731/6368, 27.18%), followed by *Managing My Symptoms* (963/6368, 15.12%). The vast majority of *My Guide* participants (35/38, 92%) accessed content related to at least one symptom within the *Managing My Symptoms* domain. Participants clicked on the least number of links within the *Breast Cancer Medications* domain (318/6368, 4.99%).

#### My Health App

Over the 6-week study period, *My Health* participants clicked on a total of 7167 links or webpages of study content within the app. The top 3 domains that participants most frequently accessed were *Diet and Nutrition* (2866/7167, 39.99%) and *Exercise* (1391/7167, 19.41%). The *Preventing Diabetes and Heart Disease* domain had the least number of links clicked (573/7167, 7.99%).

### Study Outcomes

Multimedia Appendix 3 and Multimedia Appendix 4 present the unadjusted means and score ranges of the study outcomes at each study assessment by high app use versus low app use of *My Guide* or *My Health*, respectively. For *My Guide*, scores on breast cancer well-being exceeded the minimally important difference threshold from T1 to T2 and T1 to T3 among low app users, while for *My Health*, scores on social well-being exceeded the minimally important difference threshold from T1 to T2 among high app users.

## Discussion

This study evaluated the patterns of use of 2 culturally informed, evidence-based smartphone apps designed specifically for Latina breast cancer survivors. On average, participants used their *My Guide* and *My Health* apps for 8.66 hours and 7.28 hours, respectively. In line with prior research, our study integrated strategies to improve participant engagement, including telecoaching to promote optimal adherence to app use [52], cultural relevance and sensitivity, and specific features of the smartphone apps [53] such as ease of use, design aesthetics, and mobile phone features. Additionally, Latina breast cancer survivors spent an average of more than 1 hour per week on *My Guide* or *My Health*, which is comparable to the amount of time, if not more, patients would typically spend with an in-person counselor. These findings suggest that both apps are of interest to Latina breast cancer survivors.

The past decade has seen a significant increase in technology-assisted interventions for patients with cancer [54,55]. However, due to the few culturally adapted web-based interventions for patients with cancer, evaluation of the usage of these apps among minority patients has not been well-studied. The Nuevo Amanecer smartphone app for Latina breast cancer survivors, for example, is a culturally tailored smartphone app for Latina breast cancer survivors, which focuses on physical activity promotion [56]. Results from the Nuevo Amanecer feasibility trial revealed that women checked their physical activity tracking 4-6 times per week but click level data and total minutes spent engaging in the smartphone app were not reported as part of the study findings [56]. Notably, both *My Guide* and *My Health* demonstrated longer total durations of app use over a shorter time period compared to 2 other web-based tools for breast cancer survivors in general, which reported an average of 5.6 hours [57] and 15.2 minutes [58] of user engagement across the 4-month study time frame. Our use of telecoaching and cultural tailoring of the apps may have enhanced uptake and may explain the higher use of our smartphone app relative to that reported in previously published studies.

With the exception of employment status, sociodemographic factors did not predict app use. These findings suggest that the *My Guide* and *My Health* apps are accessible to a broad group of Latina breast cancer survivors who speak English or Spanish and have varying educational and income backgrounds, age, ancestry, marital status, and baseline HRQoL. These findings also suggest that the scalability of these apps is feasible. Women who were employed were less likely to use the *My Health* app, and this finding may be explained by employed breast cancer survivors having less time to devote to the app. Results also demonstrated clinically meaningful improvements in breast cancer well-being and social well-being among low users of *My Guide* and high users of *My Health*, respectively. Descriptively, low *My Guide* users also reported reduced symptom burden over time. However, it is unclear whether these changes are clinically meaningful due to the lack of established cutoffs for minimally important differences in symptom burden. Additional research is needed to further evaluate the associations between low app use versus high app use and improvements in HRQoL subdomains as well as to determine the optimal level of weekly app use. The finding that high *My Health* users reported improved social well-being over time also warrant further evaluation, as the *My Health* app focuses on promoting general health and healthy behaviors and does not include specific content related to social support or relationships. Follow-up interviews with participants may help contextualize study results and identify specific aspects of the intervention that were beneficial in terms of study outcomes.

Click-level data revealed that users of *My Guide* most frequently accessed content related to psychosocial aspects of the cancer experience followed by physical symptoms, whereas users of *My Health* most frequently accessed content related to nutrition and exercise. These most-clicked domains are consistent with commonly documented concerns among the general population of cancer survivors [59-61], and topics related to managing emotions, managing physical symptoms, and learning healthy lifestyle behaviors after treatment completion may signify the most relevant or engaging intervention content for Latina breast cancer survivors. Providing educational materials on nutrition and exercise may be especially important as Latinas tend to have higher rates of excess body weight and obesity when compared to their non-Latina White counterparts [62,63]. Women randomized to the *My Guide* app were most likely to click on the *Managing My Emotions* module. Issues regarding coping with loss of health status, bodily changes, fear of recurrence, and reduced medical oncology visits can make the transition to survivorship an emotionally challenging time [64], which may explain the greater number of clicks within the *Managing My Emotions* module.

The results from this study should be evaluated within the context of the study limitations. First, click-level data only provide the total number of clicks for each domain and subdomain and do not account for differences in the amount of content across the domains and subdomains. For example, the most or least accessed topics may simply reflect domains that had the most or least number of links, rather than indicating which topics had more or less engagement or interest from participants. Second, rather than the total time spent using the app, another measure of app use such as intensity or depth of engagement with the intervention (eg, proportion of specific app features used out of the total available features) [65] or some other app-related factor that we did not capture may instead be associated with study outcomes. Systematic reviews on eHealth interventions have reported a variety of metrics of app usage [25,66], including measures of frequency (eg, number of logins), duration (eg, time logged in), and activity (eg, page views, modules completed), and demonstrated that intervention outcomes may be affected by different measures of eHealth usage [25]. Third, while our sample had notable sociodemographic characteristics, including being primarily Spanish-speaking, foreign-born, and Mexican ancestry, our results may not generalize to all Latina breast cancer survivors in the United States. Additionally, this study focused on Latina breast cancer survivors who completed active treatment for breast cancer within 2 years, and the differential needs of those further into survivorship may result in different intervention effects. Future research should consider the optimal timepoint within the cancer continuum of administering the intervention as it relates to the hypothesized intervention effects. Fourth, our sample size was relatively small (n=78), which limits the study findings. Lastly, our study time frame of a 6-week intervention period plus a 2-week follow-up may have been enough to establish feasibility; however, this time frame may be too short to demonstrate efficacy. Future studies should evaluate *My Guide* with a larger and more diverse sample of Latina breast cancer survivors, include other objective and comprehensive measures of participant engagement or eHealth usage (eg, frequency of use, time spent on the app, activity completion) [25,65], and examine whether app use is predictive of study outcomes over a longer study period.

In summary, this study contributes to the scarce research regarding eHealth supportive care interventions among Latina breast cancer survivors. The favorable rates of participant use across the study apps suggest that Latina breast cancer survivors are interested in the content delivered across both *My Guide* and *My Health*. Given the paucity of smartphone apps that have been developed for Latina patients, these click-level data may provide useful insights on the most important cancer topics for this historically understudied patient population. The click-level data provide information on the most accessed topics within the study apps, and these findings may lend insights into some of the most relevant survivorship topics for Latina breast cancer survivors. Furthermore, sociodemographic variables, excluding employment status, or HRQoL at study entry were not related to app use for *My Guide* and *My Health,* which suggest the potential for larger uptake among Latina breast cancer survivors. Therefore, these apps may be accessible to diverse Latina breast cancer survivors. Additional research is needed to determine the effect of eHealth use on psychosocial outcomes among Latina breast cancer survivors and whether a longer intervention time frame is needed for optimal improvements.

## Appendix

Multimedia Appendix 1 Domain descriptions for *My Guide* app and *My Health* app.

Multimedia Appendix 2 Number of clicks for each subdomain within each app domain.

Multimedia Appendix 3 Descriptive statistics of study outcomes across time for *My Guide* app.

Multimedia Appendix 4 Descriptive statistics of study outcomes across time for *My Health* app.

## Abbreviations

CASE-cancer: Communication and Attitudinal Self-Efficacy scale for cancer
FACT-B: Functional Assessment of Cancer Therapy-Breast
FACT-G7: Functional Assessment of Cancer Therapy-General 7 items
HRQoL: health-related quality of life
