# Supplementary material for: Patterns of Use of Smartphone-Based Interventions Among Latina Breast Cancer Survivors: Secondary Analysis of a Pilot Randomized Controlled Trial
Source: JMIR Cancer. 2020 Dec 8;6(2):e17538. doi: 10.2196/17538 (PMC7755528; doi:10.2196/17538)
Supplement: Multimedia Appendix 1 [file cancer_v6i2e17538_app1.docx]

**Multimedia Appendix 1*.*** Domain descriptions for *My Guide* app and *My Health* app.

| ***My Guide* app** | **Description** | **Subdomains** |
| --- | --- | --- |
| Managing My Emotions | This domain reviews ten common emotions (e.g., anxiety, stress, loneliness, sadness, relief) cancer survivors experience after treatment, and discusses relaxation and stress management strategies to improve overall mood and functioning. | Common emotions after treatment; Learning how to relax; Improving thoughts & feelings; Expressing your feelings; Improving your everyday life; Improving your relationships; Improving physical health; Reaching out to the community. |
| Managing My Symptoms | This domain reviews common physical and psychological symptoms experienced after cancer treatment, as well as methods to manage those symptoms. | Bone and joint pain, fatigue, sleep difficulties. mood symptoms. weight changes, hair loss, lymphedema, hot flashes, sex drive changes, infertility, scarring. |
| Managing My Health | This domain presents a general overview about breast cancer and adjuvant treatments, common treatment-related side effects, and lifestyle recommendations after treatment. | Breast cancer overview; Considering reconstruction; Managing side effects; Doctor recommendations; Reducing recurrence risk; Thinking of having children; Nutrition after cancer. |
| Breast Cancer Medications | This domain provides general information on hormone therapy, including different types of medications and related side effects. | Basics of hormone therapy; How to take hormone therapy; Hormone therapy side effects; Hormone therapy questions; Tips for taking hormone therapy. |
| Friends and Family | This domain discusses the ways in which cancer survivors may experience changes in their interpersonal relationships, and their roles and responsibilities at home and work following a cancer diagnosis and treatment. | Relationships; Advice for singles; Family and friendships; Changes at work; Talking to your doctor. |
| Community and Everyday Support | This domain presents relevant resources and community organizations for Latina breast cancer survivors, including Spanish support groups, health-related support, and financial assistance. | Community support; Connecting with survivors; Emotional support; Financial support; Legal support; Medical financial support; Wellness and prevention. |
|  |  |  |
| ***My Health* app** | **Description** | **Subdomains** |
| Healthy Eating | This domain highlights the importance of good nutrition and reviews which foods breast cancer survivors should eat or should limit. | Why healthy eating matters; What can I eat to stay healthy; Why is drinking water important; What foods should I limit; Myths vs. facts. |
| Eating Well | This domain discusses ways to maintain a balanced diet, including tips for eating healthy (e.g., portion control) and examples of healthy recipes. | How to keep a balanced diet; Understanding food labels; How do I keep eating healthy; Healthy recipes; Tips for healthy eating; Myths vs. facts. |
| Exercise | This domain presents an overview of the benefits of exercise and culturally appropriate approaches for increasing physical activity. | How to keep moving; Benefits of physical activity; 4 types of exercise; Benefits of salsa; Benefits of yoga; Benefits of Pilates. |
| Preventing Diabetes and Heart Disease | This domain provides general information on diabetes and heart disease prevention. | Diabetes prevention; Reduce the risks for heart disease; Myths vs. facts. |
| Lifestyle Behaviors | This domain provides recommendations for healthy lifestyle behaviors, including sun protection, tobacco cessation, handwashing, and sexual health safety. | Be safe in the sun; Stay away from tobacco; Get plenty of sleep; Importance of handwashing; Sexual health and STDs; Sexual health and Hepatitis B. |
| Doctor’s Recommendations | This domain presents guidance on medication adherence, routine doctor visits and procedures, and follow-up care. | How to take your medication; Common medication mistakes; Medication tips; See your doctor regularly; Why is follow-up care important; Importance of the flu vaccine; Understanding imaging procedures. |
