# Supplementary material for: Patterns of Use of Smartphone-Based Interventions Among Latina Breast Cancer Survivors: Secondary Analysis of a Pilot Randomized Controlled Trial
Source: JMIR Cancer. 2020 Dec 8;6(2):e17538. doi: 10.2196/17538 (PMC7755528; doi:10.2196/17538)
Supplement: Multimedia Appendix 2 [file cancer_v6i2e17538_app2.docx]

**Multimedia Appendix 2.** Number of clicks for each subdomain within each app domain.

| ***My Guide* app** | |
| --- | --- |
| Domains, subdomains | n (%), Values |
| **Managing My Emotions (n=1731)** |  |
| Emotions overview | 88 (5.08) |
| Common emotions after treatment | 429 (24.78) |
| Learning how to relax | 442 (25.53) |
| Improving thoughts & feelings | 245 (14.15) |
| Expressing your feelings | 102 (5.89) |
| Improving your everyday life | 112 (6.47) |
| Improving your relationships | 126 (7.28) |
| Improving physical health | 118 (6.82) |
| Reaching out to the community | 69 (3.99) |
| **Managing My Symptoms (n=963)** |  |
| **Managing My Health (n=784)** |  |
| Breast cancer overview | 282 (35.97) |
| Considering reconstruction? | 48 (6.12) |
| Doctor recommendations | 151 (19.26) |
| Reducing recurrence risk | 93 (11.86) |
| Thinking of having children | 23 (2.93) |
| Nutrition after cancer | 187 (23.85) |
| **Breast Cancer Medications (n=318)** |  |
| Basics of hormone therapy | 48 (15.09) |
| How to take hormone therapy | 35 (11.01) |
| Hormone therapy side effects | 78 (24.53) |
| Hormone therapy questions | 116 (36.48) |
| Tips for taking hormone therapy | 41 (12.89) |
| **Family and Friends (n=608)** |  |
| Relationships | 224 (36.84) |
| Advice for singles | 66 (10.86) |
| Family & friendships | 156 (25.66) |
| Changes at work | 91 (14.97) |
| Talking to your doctor | 71 (11.68) |
| **Community and Everyday Support (n=685)** |  |
| Community support | 92 (13.43) |
| Connecting with survivors | 108 (15.77) |
| Emotional support | 82 (11.97) |
| Financial support | 94 (13.72) |
| Legal support | 32 (4.67) |
| Medical financial support | 66 (9.64) |
| Wellness & prevention | 211 (30.80) |
| **Listen and Learn (Media; n=1279)** |  |
| Informational videos | 288 (22.52) |
| How the mind works | 394 (30.81) |
| Stories from survivors | 328 (25.65) |
| Listen to the experts | 269 (21.03) |
|  |  |
| ***My Health* app** | |
| Domains, subdomains | n (%), Values |
| **Healthy Eating (n=1339)** | 366 (27.33) |
| Why healthy eating matters | 394 (29.42) |
| What can I eat to stay healthy? | 155 (11.58) |
| Why is drinking water important? | 297 (22.18) |
| What foods should I limit? | 127 (9.48) |
| **Eat Well (n=1527)** |  |
| How to keep balanced diet | 291 (19.06) |
| Understanding food labels | 265 (17.35) |
| How do keep eating healthy? | 261 (17.09) |
| Healthy recipes | 210 (13.75) |
| Tips for healthy eating | 428 (28.03) |
| Myths vs. Facts | 72 (4.72) |
| **Exercise (n=1391)** |  |
| How to keep moving | 148 (10.64) |
| Benefits of physical activity | 126 (9.06) |
| Benefits of salsa | 115 (8.27) |
| 4 types of exercise | 390 (28.04) |
| Benefits of yoga | 330 (23.72) |
| Benefits of Pilates | 282 (20.27) |
| **Preventing Diabetes and Heart Disease (n=573)** |  |
| Overview of chronic diseases | 72 (12.57) |
| Diabetes prevention | 225 (39.27) |
| Reduce the risks for heart disease | 151 (26.35) |
| Myths vs. Facts | 125 (21.82) |
| **Lifestyle Behaviors (n=929)** |  |
| Be safe in the sun | 115 (12.38) |
| Stay away from tobacco | 93 (10.01) |
| Get plenty of sleep | 132 (14.21) |
| Importance of handwashing | 213 (22.93) |
| Sexual health and STDs | 162 (17.44) |
| Sexual health and Hepatitis B | 214 (23.04) |
| **Doctor's Recommendations (n=999)** |  |
| How to take your medication | 95 (9.51) |
| Common medication mistakes | 137 (13.71) |
| Medication tips | 103 (10.31) |
| See your doctor regularly | 135 (13.51) |
| Why is follow-up care important? | 153 (15.32) |
| Importance of the flu vaccine | 166 (16.62) |
| Understanding imaging procedures | 210 (21.02) |
| **Media (n=409)** |  |
| Healthy eating videos | 220 (53.79) |
| Videos about lifestyle behaviors | 189 (46.21) |
