# Supplementary material for: Patterns of Use of Smartphone-Based Interventions Among Latina Breast Cancer Survivors: Secondary Analysis of a Pilot Randomized Controlled Trial
Source: JMIR Cancer. 2020 Dec 8;6(2):e17538. doi: 10.2196/17538 (PMC7755528; doi:10.2196/17538)
Supplement: Multimedia Appendix 3 [file cancer_v6i2e17538_app3.docx]

**Multimedia Appendix 3.** Descriptive statistics of study outcomes across time for *My Guide* app.

|  | **High App User**  **(n=22)** | | | **Low App User**  **(n=17)** | | |
| --- | --- | --- | --- | --- | --- | --- |
|  | T1 | T2 | T3 | T1 | T2 | T3 |
| Study Outcomes | Mean (Range) | Mean (Range) | Mean (Range) | Mean (Range) | Mean (Range) | Mean (Range) |
| Physical well-being^a,f^ | 21.23  (3-28) | 21.27  (8-28) | 20.27  (6-28) | 21.18  (6-27) | 23.20 (16-28) | 21.80 (15-28) |
| Emotional well-being^a,f^ | 19.50  (5-24) | 19.09  (9-24) | 18.60  (10-24) | 18.00  (5-24) | 19.67  (13-24) | 18.87  (14-24) |
| Functional well-being^a,f^ | 20.73  (8-28) | 20.55  (10-28) | 19.95  (9-28) | 20.06  (11-26) | 20.13  (11-28) | 19.47  (7-26) |
| Social well-being^a,f^ | 21.02  (2-28) | 21.15  (8-28) | 20.90  (5-28) | 19.84  (8-28) | 20.44  (10-28) | 19.87  (7-28) |
| Breast cancer well-being^a,f^ | 23.50  (16-39) | 24.09  (14-36) | 23.41  (14-34) | 23.47  (12-36) | 26.74  (17-35) | 26.13  (14-35) |
| Symptom burden^b,g^ | 25.55  (1-57) | 23.59  (1-45) | 25.14  (4-47) | 23.47  (2-57) | 20.20  (1-31) | 21.00  (0-41) |
| Cancer-specific distress^c,g^ | 22.50  (0-67) | 25.18  (0-59) | 23.27  (1-67) | 25.71  (4-61) | 25.80  (6-57) | 20.20  (0-46) |
| Cancer-relevant self-efficacy^d,f^ | 44.05  (32-48) | 43.05  (32-48) | 43.05  (29-48) | 41.59  (31-48) | 42.53  (30-48) | 42.20  (34-48) |
| Breast cancer knowledge^e,f^ | 9.14  (4-15) | 11.32  (7-14) | 11.82  (6-16) | 9.88  (2-14) | 11.33  (8-15) | 10.47  (7-13) |

*Notes.* High app user, ≥ 60 minutes/week; Low app user, < 60 minutes/week; T1, baseline; T2, immediately after 6-week intervention; T3, 2 weeks after T2.

^a^Functional Assessment of Cancer Therapy–Breast (FACT-B); ^b^Breast Cancer Prevention Trial (BCPT); ^c^Impact of Events Scale (IES); ^d^Communication and Attitudinal Self-Efficacy scale for cancer (CASE-cancer); ^e^Knowledge about Breast Cancer questionnaire; ^f^Higher scores indicate better outcomes (i.e., domains of well-being, self-efficacy, knowledge); ^g^Higher scores indicate worse outcomes (i.e., symptom burden, cancer-specific distress).
