# Supplementary material for: Patterns of Use of Smartphone-Based Interventions Among Latina Breast Cancer Survivors: Secondary Analysis of a Pilot Randomized Controlled Trial
Source: JMIR Cancer. 2020 Dec 8;6(2):e17538. doi: 10.2196/17538 (PMC7755528; doi:10.2196/17538)
Supplement: Multimedia Appendix 4 [file cancer_v6i2e17538_app4.docx]

**Multimedia Appendix 4.** Descriptive statistics of study outcomes across time for *My Health* app.

|  | **High App User**  **(n=19)** | | | **Low App User**  **(n=20)** | | |
| --- | --- | --- | --- | --- | --- | --- |
|  | T1 | T2 | T3 | T1 | T2 | T3 |
| Study Outcomes | Mean (Range) | Mean (Range) | Mean (Range) | Mean (Range) | Mean (Range) | Mean (Range) |
| Physical well-being^a,f^ | 20.21  (2-26) | 21.56  (10-28) | 20.00  (3-28) | 21.65  (6-28) | 21.10  (4-28) | 21.00  (6-28) |
| Emotional well-being^a,f^ | 18.89  (8-24) | 18.33  (6-24) | 19.00  (7-24) | 19.00  (9-24) | 18.95  (7-24) | 17.68  (7-24) |
| Functional well-being^a,f^ | 20.42  (8-28) | 21.33  (14-28) | 21.06  (8-28) | 21.90  (13-28) | 20.55  (9-28) | 20.95  (3-28) |
| Social well-being^a,f^ | 20.74  (5-28) | 23.40  (15-28) | 22.52  (11-28) | 21.03  (4-28) | 21.64  (10-28) | 21.23  (7-28) |
| Breast cancer well-being^a,f^ | 21.37  (4-36) | 22.42  (4-36) | 22.60  (7-36) | 22.75  (7-33) | 24.20  (5-39) | 24.37  (9-38) |
| Symptom burden^b,g^ | 31.32  (8-69) | 27.39  (3-66) | 27.53  (2-59) | 24.70  (0-70) | 22.95  (0-68) | 25.37  (3-61) |
| Cancer-specific distress^c,g^ | 29.58  (0-63) | 26.56  (4-49) | 24.35  (0-49) | 17.65  (0-53) | 22.15  (0-63) | 22.05  (3-45) |
| Cancer-relevant self-efficacy^d,f^ | 43.00  (28-48) | 43.29  (30-48) | 43.81  (30-48) | 42.74  (36-48) | 41.95  (32-48) | 42.42  (35-48) |
| Breast cancer knowledge^e,f^ | 9.21  (3-13) | 9.94  (3-14) | 9.65  (4-15) | 9.35  (4-15) | 10.55  (5-14) | 10.32  (6-14) |

*Notes.* High app user, ≥ 60 minutes/week; Low app user, < 60 minutes/week; T1, baseline; T2, immediately after 6-week intervention; T3, 2 weeks after T2.

^a^Functional Assessment of Cancer Therapy–Breast (FACT-B); ^b^Breast Cancer Prevention Trial (BCPT); ^c^Impact of Events Scale (IES); ^d^Communication and Attitudinal Self-Efficacy scale for cancer (CASE-cancer); ^e^Knowledge about Breast Cancer questionnaire; ^f^Higher scores indicate better outcomes (i.e., domains of well-being, self-efficacy, knowledge); ^g^Higher scores indicate worse outcomes (i.e., symptom burden, cancer-specific distress).
